# Differentiated tumor immune microenvironment of Epstein–Barr virus-associated and negative gastric cancer: implication in prognosis and immunotherapy

**DOI:** 10.18632/oncotarget.17945

**Published:** 2017-05-16

**Authors:** Jing Ma, Jianhui Li, Yiming Hao, Yongzhan Nie, Zengshan Li, Meirui Qian, Qiaoyi Liang, Jun Yu, Musheng Zeng, Kaichun Wu

**Affiliations:** ^1^ Fourth Military Medical University, State Key Laboratory of Cancer Biology & Institute of Digestive Diseases, Xijing Hospital, Xi'an, Shaanxi, China; ^2^ Department of Infectious Diseases, Tangdu Hospital Affiliated to the Fourth Military Medical University, Xi'an, Shaanxi, China; ^3^ The Pathology Department, Fourth Military Medical University, Xi'an, Shaanxi, China; ^4^ Institute of Digestive Disease and Department of Medicine and Therapeutics, State Key Laboratory of Digestive Disease, Li Ka Shing Institute of Health Sciences, The Chinese University of Hong Kong, Hong Kong, China and The Chinese University of Hong Kong Shenzhen Research Institute, Shenzhen, Guangdong, China; ^5^ State Key Laboratory of Oncology in South China, Collaborative Innovation Center for Cancer Medicine, Department of Experimental Research, Sun Yat-sen University Cancer Center, Guangzhou, Guangdong, China

**Keywords:** gastric cancer, tumor microenvironment, Epstein-Barr virus, tumor-infiltrating lymphocytes, immune checkpoint

## Abstract

Epstein-Barr virus-associated gastric cancer (EBVaGC) has been proposed to be a distinct subtype with a specific immune microenvironment. Here, we evaluated tumor-infiltrating T-cell subsets and the expression of programmed cell death protein 1 (PD-1) and programmed death-ligand 1 (PD-L1) in 571 gastric cancers (GCs).

Tissue microarrays were stained using EBER *in situ* hybridization for EBV and using immunohistochemistry for CD4, CD8, Foxp3, PD-1 and PD-L1. GCs were categorized into four types based on CD8^+^ infiltration and PD-L1 expression. The 5-year overall survival (OS) was evaluated according to EBV infection, T-cell subsets, PD-1 and PD-L1 expression and immune types.

Thirty-two (5.3%) EBVaGCs were identified, which were more prevalent for CD8^+^ (p<0.001) and Foxp3^+^ (p=0.020) cell infiltration than EBV-negative GCs (EBVnGCs), suggesting a better 5-year OS (p=0.003). CD8^+^ (p=0.001) and Foxp3^+^ (p=0.018) cell infiltration was associated with better 5-year OS, whereas PD-L1 expression correlated with a poor 5-year OS (p=0.002). EBVaGC and EBVnGC had heterogeneous immune microenvironment, with CD8^+^ PD-L1^−^ GC exhibiting the best 5-year OS (p<0.001).

GC was an immune ignorant dominant tumor and poor to no T-cell infiltration. An immune type classification algorithm can provide prognostic information and a rational basis for immunotherapy.

## INTRODUCTION

Epstein-Barr virus (EBV) is a member of the Herpesviridae family that latently infect greater more than 90% of adults worldwide and is associated with several human malignancies, such as Hodgkin's lymphoma, Burkitt's lymphoma, nasal NK/T-cell lymphoma, nasopharyngeal carcinoma and a subset of gastric cancer (GC) [1]. Recently, the Cancer Genome Atlas Research Network published a comprehensive molecular characterization of gastric adenocarcinoma and classified EBV-associated GC (EBVaGC) as a distinct GC type that is characterized by recurrent PIK3CA mutations, extreme DNA hypermethylation, and amplification of JAK2 and programmed death ligand-1/2 (PD-L1/PD-L2) [2]. A meta-analysis demonstrated that the prognosis for EBVaGC was better than that for EBV-negative GC (EBVnGC) [3], although, the underlying mechanisms of this effect are not clear. EBVaGC often features extensive lymphocytes infiltration [4], especially CD8^+^ (cluster of differentiation 8) T cells, which might have a cytotoxic effect on EBV-positive tumor cells [5].

As a “non-self” component, tumor cells can trigger immune responses characterized by the infiltration of various immune cells, which can affect tumor progression. In addition, the tumor develops many strategies to evade an immune response, including immune suppressors such as regulatory T cells (Treg) and myeloid-derived suppressor cells (MDSC) [6], immune checkpoint cytotoxic T-lymphocyte-associated protein 4 (CTLA-4), programmed death protein 1 (PD-1)/PD-L1 [7] and T cell dysfunction [8]. Based on the presence or absence of tumor-infiltrating lymphocytes (TILs) and PD-L1 expression, four tumor immune microenvironment types have been proposed [9], including type I (TILs^+^ PD-L1^+^ associated with adaptive immune resistance), type II (TILs^−^ PD-L1^−^ indicating immune ignorance), type III (TILs^−^ PD-L1^+^ indicating intrinsic induction) and type IV (TILs^+^ PD-L1^−^ indicating other suppressors promoting immune tolerance). To some extent, this stratification has prognostic value and indicates possible cancer immunotherapy strategies.

In this study, we conducted a retrospective analysis to evaluate EBV infection status, tumor-infiltrating T-cell subsets and PD-1/PD-L1 expression in 571 tumor samples. Furthermore, we classified these tumor samples into four cancer types defined by CD8/PD-L1 status. Overall survival (OS) was analyzed according to EBV infection, T-cell subsets, PD-1/PD-L1 expression and cancer types.

## RESULTS

### Clinicopathological GC features

**Figure 1:**
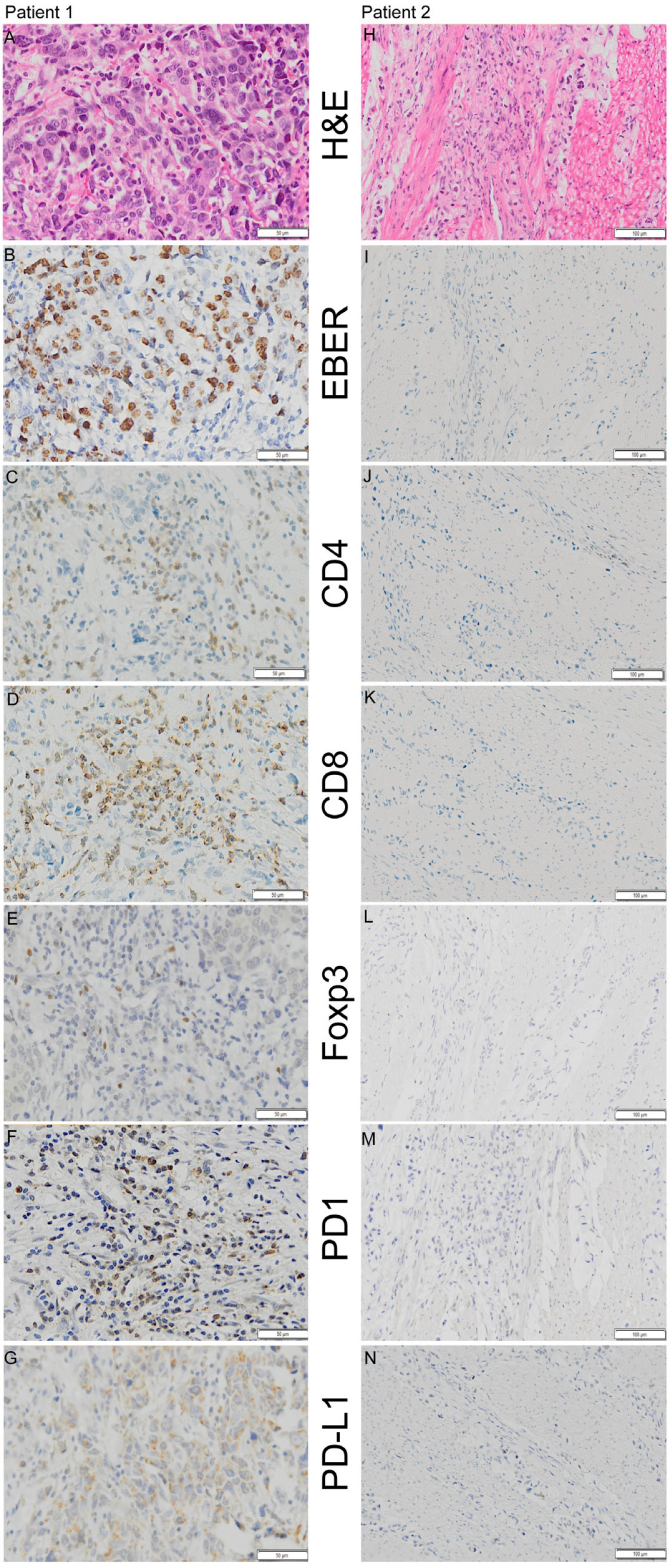
Representative examples of H&E staining, EBER (Epstein-Barr Virus-encoded RNA) *in situ* hybridization and CD4 (cluster of differentiation 4), CD8 (cluster of differentiation 8), Foxp3 (Forkhead box P3), PD-1 (programmed death 1), and PD-L1 (programmed death ligand-1) immunohistochemistry staining in 2 patients Patient 1 **(A, B, C, D, E, F and G)** was positive for EBER, CD4, CD8, Foxp3, PD-1 and PD-L1 (Shown at ×200 original magnification), whereas patient 2 **(H, I, J, K, L, M and N)** was negative for EBER, CD4, CD8, Foxp3, PD-1 and PD-L1 (shown at ×100 original magnification).

The clinicopathological features of 571 patients were described in Supplementary Table 1. H&E staining (Figure 1A, 1H), EBER (Figure 1B, 1I), CD8 (Figure 1D, 1K) and PD-L1 (Figure 1G, 1N) were evaluated in all samples, and CD4 (cluster of differentiation 4) (Figure 1C, 1J), Foxp3 (Forkhead box P3) (Figure 1E, 1L) and PD-1 (Figure 1F, 1M) were evaluated in 567 (99.3%), 559 (97.9%) and 522 (91.4%) samples, respectively. The unmeasured samples resulted from core loss during IHC staining. EBV infection was detected in 32 (5.3%) samples using EBER *in situ* hybridization. There was no significant difference in gender, AJCC stage, tumor location, depth, histological classification and differentiation between EBVaGC and EBVnGC. The age of patients with EBVaGC (median 54, range 31-72) was younger than that of patients with EBVnGC (median 59, range 21-87, p=0.049).

### OS, T-cell infiltration subsets and PD-1/PD-L1 expression between EBVaGC and EBVnGC cohorts

**Figure 2:**
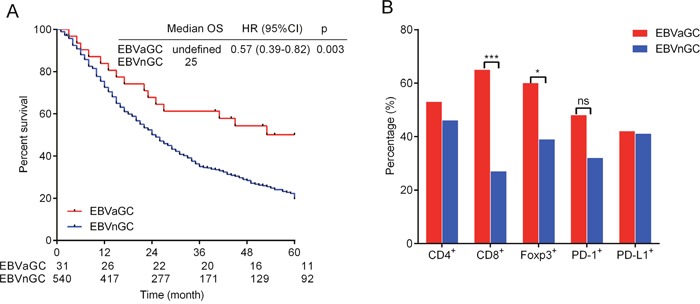
Kaplan-Meier method log-rank testing demonstrated a better 5-year overall survival (OS) in EBVaGC (Epstein-Barr Virus-associated gastric cancer) than in EBVnGC (Epstein-Barr Virus-negative gastric cancer) **(A)**. Percentage of CD4 (cluster of differentiation 4), CD8 (cluster of differentiation 8) and Foxp3 (Forkhead box P3) positive cells and PD-1 (programmed death 1), PD-L1 (programmed death ligand-1) expression in EBVaGC and EBVnGC **(B)**. Tow-tailed Pearson χ2 and Fisher's exact test were used.

Fifteen patients (48.4%) in the EBVaGC cohort and 410 patients (75.9%) in the EBVnGC cohort died during the 5-year follow-up. Five patients in the EBVaGC cohort and 49 patients in the EBVnGC cohort were lost to follow-up. The log rank test indicated a better OS in the EBVaGC cohort than in the EBVnGC cohort (p=0.003, Figure 2A).

CD8^+^ and Foxp3^+^ cell infiltration were more prevalent in EBVaGC than in EBVnGC (CD8^+^ 64.5% vs. 27.2%, p<0.001 and Foxp3^+^ 60.0% vs. 38.6%, p=0.020). The CD4^+^ cell infiltration frequency was 46.0% in EBVnGC, which was lower than but no significantly different from the 53.3% in EBVaGC (p=0.433). PD-1 and PD-L1 expression in EBVaGC and EBVnGC were 48.00% and 32.06% (p=0.092) and 41.94% and 40.93% (p=0.912), respectively, and these values were not significant different (Figure 2B).

### T-cell infiltration subsets, PD-1/PD-L1 expression and OS correlation in GC

The presence of CD4^+^, CD8^+^ and Foxp3^+^ cell infiltration was 46.4% (263/567), 29.3% (167/571) and 39.7% (222/559) in the complete cohort, respectively. PD-1/PD-L1 expression was 32.8% (171/522) and 40.98% (221/571). Both the presence of Foxp3 (p=0.044) and PD-1 expression (p<0.001) were associated with CD8^+^ cell infiltration, whereas the presence of Foxp3^+^ cells(p=0.003), PD-1 expression (p<0.001) and PD-L1 expression (p=0.014) were associated with CD4^+^ cell infiltration. Moreover, PD-L1 expression was associated with PD-1 expression (p=0.037, Table 1).

**Table 1:** Interrelationship of tumor immune microenvironment features

Parameter	PD-L1 expression			PD-1 expression			Foxp3		
	PD-L1(−) n (%)	PD-L1(+) n (%)	p^#^	PD-1(−) n (%)	PD-1(+) n (%)	p^#^	Foxp3(−) n (%)	Foxp3(+) n (%)	p^#^
CD4	567			519			555		
Absent	194(63.8)	110(36.2)	0.014	224(81.5)	51(18.5)	<0.001	193(66.1)	99(33.9)	0.003
Present	141(53.6)	122(46.4)		124(50.8)	120(49.2)		141(53.6)	122(46.4)	
CD8	571			523			559		
Absent	244(60.4)	160(39.6)	0.298	278(76.6)	85(23.4)	<0.001	247(63.0)	145(37.0)	0.044
Present	93(55.7)	74(44.3)		74(46.2)	86(53.8)		90(53.9)	77(46.1)	
PD-1	522								
Absent	220(62.5)	132(37.5)	0.037						
Present	90(52.9)	80(47.1)							

# Pearson χ2 or Fisher's exact test, two-side

Patients with CD4^+^ (p=0.001), CD8^+^ (p<0.001) and Foxp3^+^ (p=0.001) cell infiltration exhibited a better 5-year OS (Table 2). Patients with PD-L1 expression had a worse 5-year OS (p=0.042, Table 2), although differences were not observed for PD-1 expression (p=0.364, Table 2 and Supplementary Figure 1). In the above analyses, clinicopathological features were indiscriminate between the groups (Supplementary Table 2). Cox analysis demonstrated that CD8^+^ and Foxp3^+^ cell infiltration predicted better survival, whereas PD-L1 expression was a risk factor in the complete cohort (Table 2). In EBVaGC, CD8^+^ cell infiltration predicted a better 5-year OS ([HR] was 0.18, 95%CI was 0.05 to 0.61, p=0.006. Supplementary Figure 2), whereas other parameters had no such prognostic value (data were not shown).

**Table 2:** Univariate and multivariate analysis of five immune-related factors

	Univariate analysis		Multivariate analysis	
	HR (95%CI)	Sig	HR (95%CI)	Sig
CD4	0.73 (0.60-0.88)	0.001	0.88 (0.71-1.09)	0.247
CD8	0.63 (0.51-0.77)	<0.001	0.66 (0.52-0.85)	0.001
Foxp3	0.72 (0.59-0.88)	0.001	0.74 (0.60-0.91)	0.004
PD-L1	1.23 (1.01-1.50)	0.042	1.27 (1.04-1.55)	0.018
PD-1	1.10 (0.89-1.37)	0.364		

Abbreviations: HR: Hazard ratio; CI: confidence interval.

We also analyzed the CD4^+^, CD8^+^ and Foxp3^+^ cell infiltration and PD-1/PD-L1 expression based on TNM stage. With tumor TNM stage advanced, less CD4^+^, CD8^+^ and Foxp3^+^ cell infiltration and PD-1 expression was found in the complete cohort (Supplementary Figure 3).

### Classification of the tumor immune microenvironment based on CD8^+^ cell infiltration and PD-L1 expression

**Figure 3:**
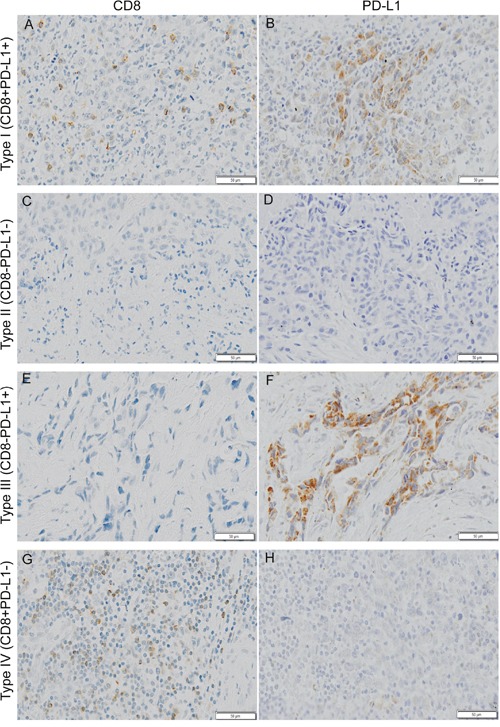
Typical examples of four types of tumor immune microenvironment based on CD8 (cluster of differentiation 8) and PD-L1 (programmed death ligand 1) immunohistochemistry staining Shown at ×100 original magnification.

**Figure 4:**
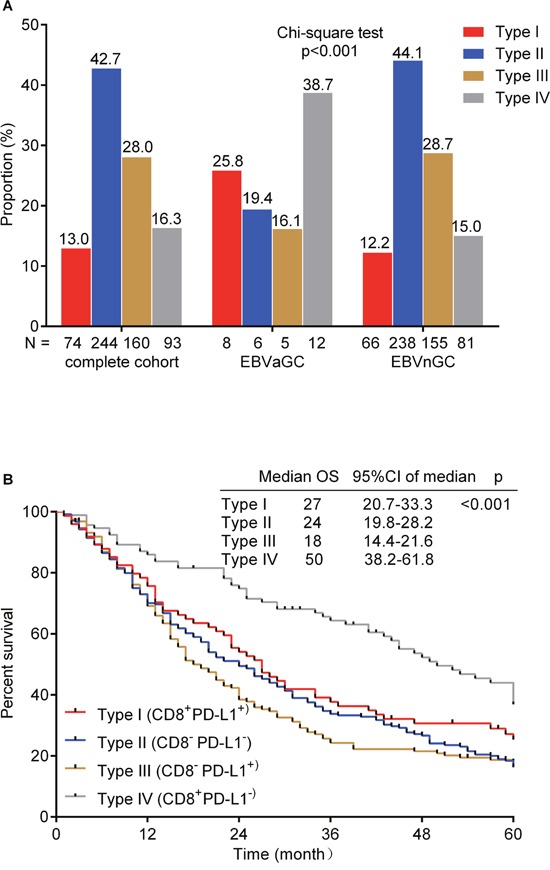
Distribution of the four tumor immune microenvironment types in gastric cancer **(A)**. The number of patients in each cohort (complete cohort, EBVaGC and EBVnGC) is under the X-axis and the proportion of four types in each cohort is labelled above the bar. Chi-square test was used. Kaplan-Meier method log-rank testing for 5-year overall survival (OS) of the four tumor immune microenvironment types **(B)**. The median OS, 95% confidence interval (CI) of median OS and p value are at the top-right corner.

To identify the combined prognostic ability of CD8^+^ cell infiltration and PD-L1 expression, we categorized 571 gastric cancers into four different tumor microenvironments based on CD8^+^ cell infiltration and PD-L1 expression (Figure 3). The distribution of the four tumor immune microenvironment types was 12.96% (type I, CD8^+^PD-L1^+^), 42.73% (type II, CD8^−^PD-L1^−^), 28.02% (type III, CD8^−^PD-L1^+^) and 16.28% (type IV, CD8^+^PD-L1^−^) in the complete cohort. To be specific, the proportion was 25.81% and 12.22% (type I), 19.35% and 44.07% (type II), 16.13% and 28.70% (type III) and 38.71% and 15.00% (type IV) in EBVaGC and EBVnGC, respectively, showing a significant difference between them (p<0.001, Figure 4A). The log rank test demonstrated that type IV had the best 5-year OS in the complete cohort (p<0.001, Figure 4B).

## DISCUSSION

In this study, we determined the EBV status of 571 GC patients and profiled tumor-infiltrating T-cell subsets and PD-1/PD-L1 expression patterns. The frequency of EBV infection in GC was 5.3%, and most patients came from northern and western China. The lower frequency of EBV infection in our study might be because of geographical and environmental factors [3, 10, 11], and EBV infection was associated with younger age.

Several studies have demonstrated that EBVaGC has a better clinical outcome which agrees with the results of our study [3, 4, 12]. In our study, EBVaGC was predominantly characterized by CD8^+^ and Foxp3^+^ cell infiltration, and in the complete cohort, more frequent infiltration of CD8^+^ and Foxp3^+^ cells correlated with a better OS. Therefore, we speculated that the adaptive immune response might have a positive effect on EBVaGC prognosis. EBVaGC displays a latency I/II pattern of EBV gene expression, typically characterized by LMP2 and EBNA1 expression [13, 14]. EBV-associated proteins and some mutated tumor cells proteins can elicit EBV-specific and tumor antigen-specific cytotoxic T lymphocytes, which can eradicate EBV-positive malignant cells [15–17].

PD-1 is expressed on activated T and B cells whereas PD-L1 is generally expressed on many immune cells, including macrophages and dendritic cells, and can be induced by inflammatory cytokines in tumor cells [7]. PD-L1 expression has been observed in various malignancies and is a major factor used to evaluate therapy responses to anti-PD-1 [18–21]. We found relatively more frequent PD-1 expression and comparable PD-L1 expression in EBVaGC compared with that in EBVnGC. Based on CD8^+^ cell infiltration and PD-L1 expression, EBVaGCs were primarily type I and type IV cancers, suggesting a favorable immune microenvironment, whereas most EBVnGCs were type II and type III cancers, which indicated that most EBVnGCs did not naturally induce effector T-cell responses. Moreover, we demonstrated that type IV GC had the best 5-year OS, which might explain why EBVaGC has a better prognosis, although immune checkpoint blockade has overall been a disappointing GC treatment. Tumor regression after therapeutic PD-1 blockade requires pre-existing CD8^+^ T cells that are negatively regulated by PD-1/PD-L1 expression [22]. Hence, immune checkpoint inhibitors treatments in type I cancer might partially relieve adaptive immune resistance and exert a positive effect on clinical outcome. Considering the lack of T cell infiltration in type II and type III GCs, a T-cell inducing agent such as a tumor vaccine combined with immune checkpoint inhibitors might improve GC survival.

A recent study demonstrated that increasing CD8^+^ T cell infiltration and PD-L1 expression were associated with impaired progression-free survival and OS in 34 gastric adenocarcinomas, which contradicts findings that TILs are associated with better disease outcomes in GC [23–25]. In our study, CD8^+^ cell infiltration predicted a better 5-year OS, whereas PD-L1 expression predicted a poor prognosis. Moreover, we found that Foxp3^+^ cell infiltration and PD-1 expression were associated with CD4^+^ and CD8^+^ cell infiltration, and the same correlation was observed between PD-L1 expression and CD4^+^ cell infiltration, but not between PD-L1 expression and CD8^+^ cell infiltration. Furthermore, PD-L1 expression was significantly associated with PD-1 expression. A comparable study illustrated that PD-L1 expression was significantly associated with infiltrating immune cells and PD-1 expression [26]. The expression of immunosuppressive markers and the presence of immune inhibitory cells do not indicate ineffective anti-tumor immune response, but are indicators of an immune response [27], because immune suppression usually occurs after immune activation.

Our study had several limitations. We had only 32 EBVaGC cases, which restricts T cell subset infiltration and PD-1/PD-L1 expression analysis in this subgroup. We did not find an association between PD-L1 expression and CD8^+^ cell infiltration, which was observed in other studies, and requires further investigation. Tissue microarray IHC staining as a high throughput technique can facilitate this type of study, but bias might still arise because only a relatively small tissue area was deliberately selected after H&E staining confirmation. Moreover, a tissue microarray with only a 1.5mm diameter was not suitable for quantitative evaluation of lymphocytes infiltrating in tumor and immune stroma, and our results were qualitative. This is a retrospective study, and all of the results are based on formalin-fixed, paraffin-embedded tissue and should be validated on live cells and in clinical trials.

In summary, this study showed that there were more frequent tumor-infiltrating CD8^+^ and Foxp3^+^ cells in EBVaGC, which predicted a better OS in this subpopulation. We first categorized GCs into four different tumor microenvironment types based on the presence or absence of CD8^+^ cells and PD-L1 expression in a large cohort (n=571). EBVaGC and EBVnGC showed heterogeneous immune microenvironments. Approximately 65% of EBVaGCs presented with a type I or type IV microenvironment, suggesting activated adaptive immune responses and a relatively better clinical outcome, whereas more than 70% of EBVnGCs were type II and type III cancers, indicating a lack of immune response and a poor prognosis. A low level of TILs might partially explain the high mortality of GC and restrict the use of antibodies that target immune checkpoints. We hope that this immune microenvironment classification algorithm can provide a rational basis for guiding cancer immunotherapy in future clinical practice.

## MATERIALS AND METHODS

### Patients and specimens

GC specimens were collected from 608 patients who received primary tumor surgical excision at Xijing Digestive Hospital between Jan 2007 and Jan 2010. All of the patients with primary GCs were included unless they had any of following conditions: previous chemotherapy prior to surgery, longstanding autoimmune disease and/or glucocorticoid treatment, HIV positivity, lymphoma or other sources of gastric metastatic carcinoma. The tissue microarray method was applied (a 1.5-mm diameter column of tissue core). Age, sex, the American Joint Committee on Cancer (AJCC) system for TNM stage (T: depth of tumor invasion, N: lymph node metastasis, M: distant metastasis), tumor location, depth, histological classification and differentiation were assessed.

All patients were followed for 5 years. In analysis, we excluded one gastric lymphoma, and 36 patients were lost at the first follow-up. In the end, 571 patients were analyzed and evaluated.

### *In situ* hybridization (ISH) for EBV-encoded small RNA (EBER)

Tissue microarrays including 608 specimens were used for EBER *in situ* hybridization (ISH). EBER, a non-coding RNA, is the most abundant RNA in EBV-infected gastric tumor cells. 4μm thick sections were cut, deparaffinized and dehydrated. EBERs in tissue microarrays of primary tumor specimens were hybridized using an Epstein Barr virus Probe ISH Kit, and were detected with the HRP/DAB Detection System (ISH5021, PanPath, Amsterdam, Netherlands) according to the manufacturer's protocols. Cases with strong signals within tumor cells were considered EBV positive. The results were determined by two independent pathologists. If they did not agree the same, then the third pathologist participated in the decision.

### Immunohistochemistry

Formalin-fixed, paraffin-embedded tissue microarrays were cut into 4μm thick sections and processed for IHC staining. Sections were deparaffinized and dehydrated with serial passages through changes of xylenes and graded ethanol. All of the microarrays were subjected to hear-induced epitope retrieval for 10 min at 100°C (citric acid sodium citrate solution was used for CD4, CD8, Foxp3 (Forkhead box P3), the pH was 6.0, and universal HIER antigen retrieval reagent was used for PD-L1, Abcam). Endogenous tissue peroxidase was blocked by slide incubation in 3% hydrogen peroxide solution for 10 min at room temperature prior to incubation with blocking serum 30 min at room temperature. Subsequently, the microarrays were incubated with primary antibodies (anti-Foxp3, 1:200, Abcam, which was diluted by antibody diluent, Abcam) overnight at 4°C, 4 hours at 37°C for PD-L1 (10μg/ml, Abcam) and 2 hours at room temperature for CD4 and CD8 with no dilutions. Goat anti-mouse/rabbit secondary antibody and HRP-labeled biotin were used to bind primary antibody. Antigen-antibody binding was visualized via application of 3.3’ diaminobenzidine (DAB) chromogen. PD-1 (Polyclonal Goat IgG, 5μg/ml, R&D) IHC was achieved using the anti-goat HRP-DAB Cell & Tissue Staining Kit (brown, R&D) according to the manufacturer's protocols. A negative control was obtained by using a normal rabbit/mouse IgG. Stained sections were counterstained with hematoxylin and coverslipped for review.

### IHC analysis

Tissue microarrays were scanned under ×100 and ×200 magnification using Olympus Soft Imaging Solutions GmbH. The levels of CD4, CD8 and Foxp3 expression were defined as follows: cases were scored at 5% intervals for CD4 and CD8, which meant that specimens with≥5% membranous expression were considered positive. The presence of PD-L1 was estimated according to the same criteria [26]. Positive Foxp3 infiltration is defined by a greater than 1% presence of Foxp3^+^ cell relative to the total cell number. PD-1 expression was positive when≥1% of lymphocytes showed membrane staining. Based on the presence or absence of CD8^+^ cell infiltration and PD-L1 expression, tumor immune microenvironment was classified as follows: type I (CD8^+^PD-L1^+^), type II (CD8^−^PD-L1^−^), type III (CD8^−^PD-L1^+^) and type IV (CD8^+^PD-L1^−^). The results were determined by two independent pathologists. If they did not agree the same, then the third pathologist participated in the decision.

### Statistical analysis

Categorical data were compared using χ2 test or Fisher's exact test with a two-sided p value. Bonferroni correction for multiple comparisons was applied when necessary. Age was described using median and minimum/maximum values and analyzed using the Mann-Whitney U test. Survival curves were constructed by using the Kaplan-Meier method and compared by using a log-rank test. Hazard ratios (HRs) and 95% confidence interval (95%CI) for 5-year survival by EBV infection and CD4^+^, CD8^+^, Foxp3^+^ cell infiltration and PD-1/PD-L1 expression were estimated in Cox regression models. Five-year overall survival (OS) was defined from the date of surgery to the date of death from any cause, or to month 60 if the patient is alive. The Cox proportional hazard regression model was used to define independent prognostic biomarkers such as CD4, CD8, Foxp3 and PD-L1 that inversely impacted OS. Statistical analyses and graphics were obtained using SPSS 21.0 software (Chicago, USA) and Graph pad 5.0. A two-tailed p<0.05 was considered statistically significant.

## SUPPLEMENTARY MATERIALS FIGURES AND TABLES







## Abbreviations

AJCC: American Joint Committee on Cancer
CD4: cluster of differentiation 4
CD8: cluster of differentiation 8
CTLA-4: cytotoxic T-lymphocyte-associated protein 4
CI: confidence interval
EBV: Epstein-Barr Virus
EBVaGC: Epstein-Barr virus-associated gastric cancer
EBVnGC: Epstein-Barr virus-negative gastric cancer
EBNA1: Epstein–Barr nuclear antigen 1
Foxp3: Forkhead box P3
GC: gastric cancer
H&E: hematoxylin-eosin staining
HR: Hazard Ratio
ISH: *in situ* hybridization
IHC: immunohistochemistry
LMP2: latent membrane protein 2
OS: overall survival
PD-1: programmed cell death protein 1
PD-L1: programmed death-ligand 1
TILs: tumor-infiltrating lymphocytes.

## References

[1] Young LS, Rickinson AB (2004). Epstein-Barr virus: 40 years on. Nat Rev Cancer.

[2] Bass AJ, Thorsson V, Shmulevich I, Reynolds SM, Miller M, Bernard B, Hinoue T, Laird PW, Curtis C, Shen H, Weisenberger DJ, Schultz N, Shen R, Cancer Genome Atlas Research Network (2014). Comprehensive molecular characterization of gastric adenocarcinoma. Nature.

[3] Camargo MC, Kim WH, Chiaravalli AM, Kim KM, Corvalan AH, Matsuo K, Yu J, Sung JJ, Herrera-Goepfert R, Meneses-Gonzalez F, Kijima Y, Natsugoe S, Liao LM (2014). Improved survival of gastric cancer with tumour Epstein-Barr virus positivity: an international pooled analysis. Gut.

[4] Song HJ, Srivastava A, Lee J, Kim YS, Kim KM, Ki Kang W, Kim M, Kim S, Park CK, Kim S (2010). Host inflammatory response predicts survival of patients with Epstein-Barr virus-associated gastric carcinoma. Gastroenterology.

[5] van Beek J, zur Hausen A, Snel SN, Berkhof J, Kranenbarg EK, van de Velde CJ, van den Brule AJ, Middeldorp JM, Meijer CJ, Bloemena E (2006). Morphological evidence of an activated cytotoxic T-cell infiltrate in EBV-positive gastric carcinoma preventing lymph node metastases. Am J Surg Pathol.

[6] Gabrilovich DI, Ostrand-Rosenberg S, Bronte V (2012). Coordinated regulation of myeloid cells by tumours. Nat Rev Immunol.

[7] Pardoll DM (2012). The blockade of immune checkpoints in cancer immunotherapy. Nat Rev Cancer.

[8] Fourcade J, Sun Z, Pagliano O, Guillaume P, Luescher IF, Sander C, Kirkwood JM, Olive D, Kuchroo V, Zarour HM (2012). CD8(+) T cells specific for tumor antigens can be rendered dysfunctional by the tumor microenvironment through upregulation of the inhibitory receptors BTLA and PD-1. Cancer Res.

[9] Teng MW, Ngiow SF, Ribas A, Smyth MJ (2015). Classifying Cancers Based on T-cell Infiltration and PD-L1. Cancer Res.

[10] van Beek J, zur Hausen A, Klein Kranenbarg E, van de Velde CJ, Middeldorp JM, van den Brule AJ, Meijer CJ, Bloemena E (2004). EBV-positive gastric adenocarcinomas: a distinct clinicopathologic entity with a low frequency of lymph node involvement. J Clin Oncol.

[11] Wakiguchi H (2002). Overview of Epstein-Barr virus-associated diseases in Japan. Crit Rev Oncol Hematol.

[12] Shinozaki-Ushiku A, Kunita A, Fukayama M (2015). Update on Epstein-Barr virus and gastric cancer (review). Int J Oncol.

[13] Liang Q, Yao X, Tang S, Zhang J, Yau TO, Li X, Tang CM, Kang W, Lung RW, Li JW, Chan TF, Xing R, Lu Y, Lo KW, Wong N, To KF (2014). Integrative identification of Epstein-Barr virus-associated mutations and epigenetic alterations in gastric cancer. Gastroenterology.

[14] zur Hausen A, Brink AA, Craanen ME, Middeldorp JM, Meijer CJ, van den Brule AJ (2000). Unique transcription pattern of Epstein-Barr virus (EBV) in EBV-carrying gastric adenocarcinomas: expression of the transforming BARF1 gene. Cancer Res.

[15] Kim SY, Park C, Kim HJ, Park J, Hwang J, Kim JI, Choi MG, Kim S, Kim KM, Kang MS (2015). Deregulation of immune response genes in patients with Epstein-Barr virus-associated gastric cancer and outcomes. Gastroenterology.

[16] Lee HS, Chang MS, Yang HK, Lee BL, Kim WH (2004). Epstein-barr virus-positive gastric carcinoma has a distinct protein expression profile in comparison with epstein-barr virus-negative carcinoma. Clin Cancer Res.

[17] Kuzushima K, Nakamura S, Nakamura T, Yamamura Y, Yokoyama N, Fujita M, Kiyono T, Tsurumi T (1999). Increased frequency of antigen-specific CD8(+) cytotoxic T lymphocytes infiltrating an Epstein-Barr virus-associated gastric carcinoma. J Clin Invest.

[18] Ansell SM, Lesokhin AM, Borrello I, Halwani A, Scott EC, Gutierrez M, Schuster SJ, Millenson MM, Cattry D, Freeman GJ, Rodig SJ, Chapuy B, Ligon AH (2015). PD-1 blockade with nivolumab in relapsed or refractory Hodgkin's lymphoma. N Engl J Med.

[19] Garon EB, Rizvi NA, Hui R, Leighl N, Balmanoukian AS, Eder JP, Patnaik A, Aggarwal C, Gubens M, Horn L, Carcereny E, Ahn MJ, Felip E, KEYNOTE-001 Investigators (2015). Pembrolizumab for the treatment of non-small-cell lung cancer. N Engl J Med.

[20] Motzer RJ, Escudier B, McDermott DF, George S, Hammers HJ, Srinivas S, Tykodi SS, Sosman JA, Procopio G, Plimack ER, Castellano D, Choueiri TK, Gurney H, CheckMate 025 Investigators (2015). Nivolumab versus Everolimus in Advanced Renal-Cell Carcinoma. N Engl J Med.

[21] Armand P, Nagler A, Weller EA, Devine SM, Avigan DE, Chen YB, Kaminski MS, Holland HK, Winter JN, Mason JR, Fay JW, Rizzieri DA, Hosing CM (2013). Disabling immune tolerance by programmed death-1 blockade with pidilizumab after autologous hematopoietic stem-cell transplantation for diffuse large B-cell lymphoma: results of an international phase II trial. J Clin Oncol.

[22] Tumeh PC, Harview CL, Yearley JH, Shintaku IP, Taylor EJ, Robert L, Chmielowski B, Spasic M, Henry G, Ciobanu V, West AN, Carmona M, Kivork C (2014). PD-1 blockade induces responses by inhibiting adaptive immune resistance. Nature.

[23] Thompson ED, Zahurak M, Murphy A, Cornish T, Cuka N, Abdelfatah E, Yang S, Duncan M, Ahuja N, Taube JM, Anders RA, Kelly RJ (2016). Patterns of PD-L1 expression and CD8 T cell infiltration in gastric adenocarcinomas and associated immune stroma. Gut.

[24] Lee HE, Chae SW, Lee YJ, Kim MA, Lee HS, Lee BL, Kim WH (2008). Prognostic implications of type and density of tumour-infiltrating lymphocytes in gastric cancer. Br J Cancer.

[25] Ishigami S, Natsugoe S, Tokuda K, Nakajo A, Xiangming C, Iwashige H, Aridome K, Hokita S, Aikou T (2000). Clinical impact of intratumoral natural killer cell and dendritic cell infiltration in gastric cancer. Cancer Lett.

[26] Taube JM, Klein A, Brahmer JR, Xu H, Pan X, Kim JH, Chen L, Pardoll DM, Topalian SL, Anders RA (2014). Association of PD-1, PD-1 ligands, and other features of the tumor immune microenvironment with response to anti-PD-1 therapy. Clin Cancer Res.

[27] Schalper KA, Velcheti V, Carvajal D, Wimberly H, Brown J, Pusztai L, Rimm DL (2014). in situ tumor PD-L1 mRNA expression is associated with increased TILs and better outcome in breast carcinomas. Clin Cancer Res.

