# Supplementary material for: Differentiated tumor immune microenvironment of Epstein–Barr virus-associated and negative gastric cancer: implication in prognosis and immunotherapy
Source: Oncotarget. 2017 May 16;8(40):67094–103. doi: 10.18632/oncotarget.17945 (PMC5620158; doi:10.18632/oncotarget.17945)
Supplement: Supplementary file 1 [file oncotarget-08-67094-s001.pdf]

Differentiated tumor immune microenvironment of Epstein–Barr virus-associated and negative gastric cancer: implication in prognosis and immunotherapy

SUPPLEMENTARY MATERIALS

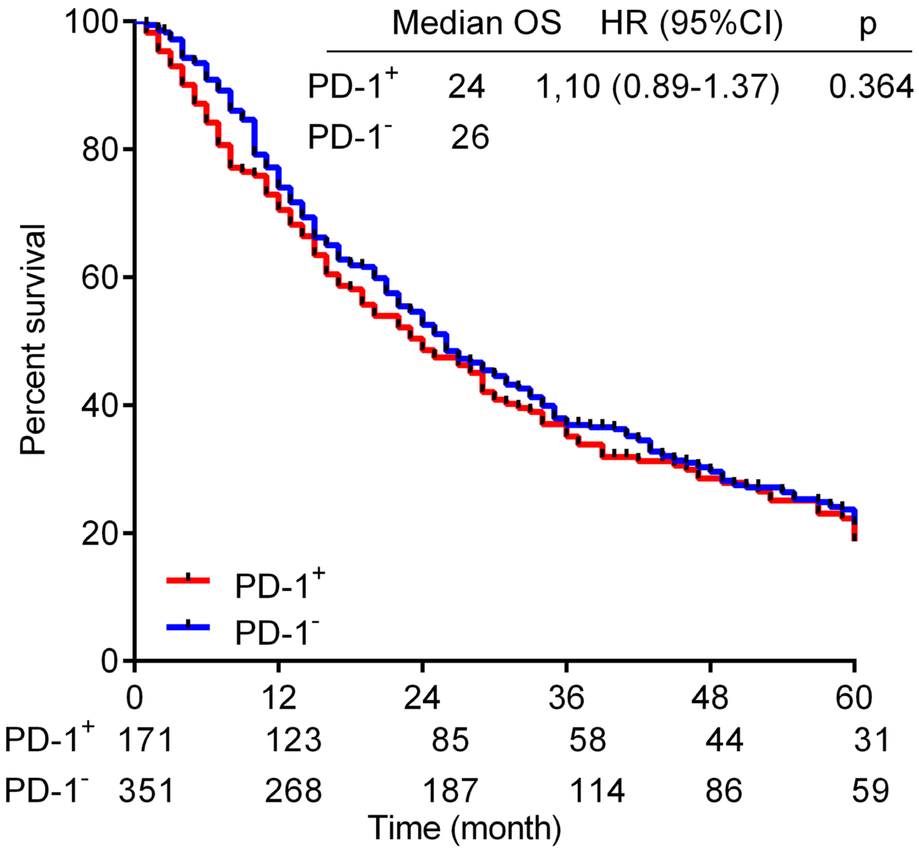

Supplementary Figure 1: Kaplan-Meier method log-rank testing for 5-year overall survival (OS) by Programmed death 1 (PD-1) expression in gastric cancer. PD-1 expression had no correlation with 5-year OS in complete cohort. ([HR] was 1.10, 95% confidence interval was 0.89 to 1.37, p=0.364).

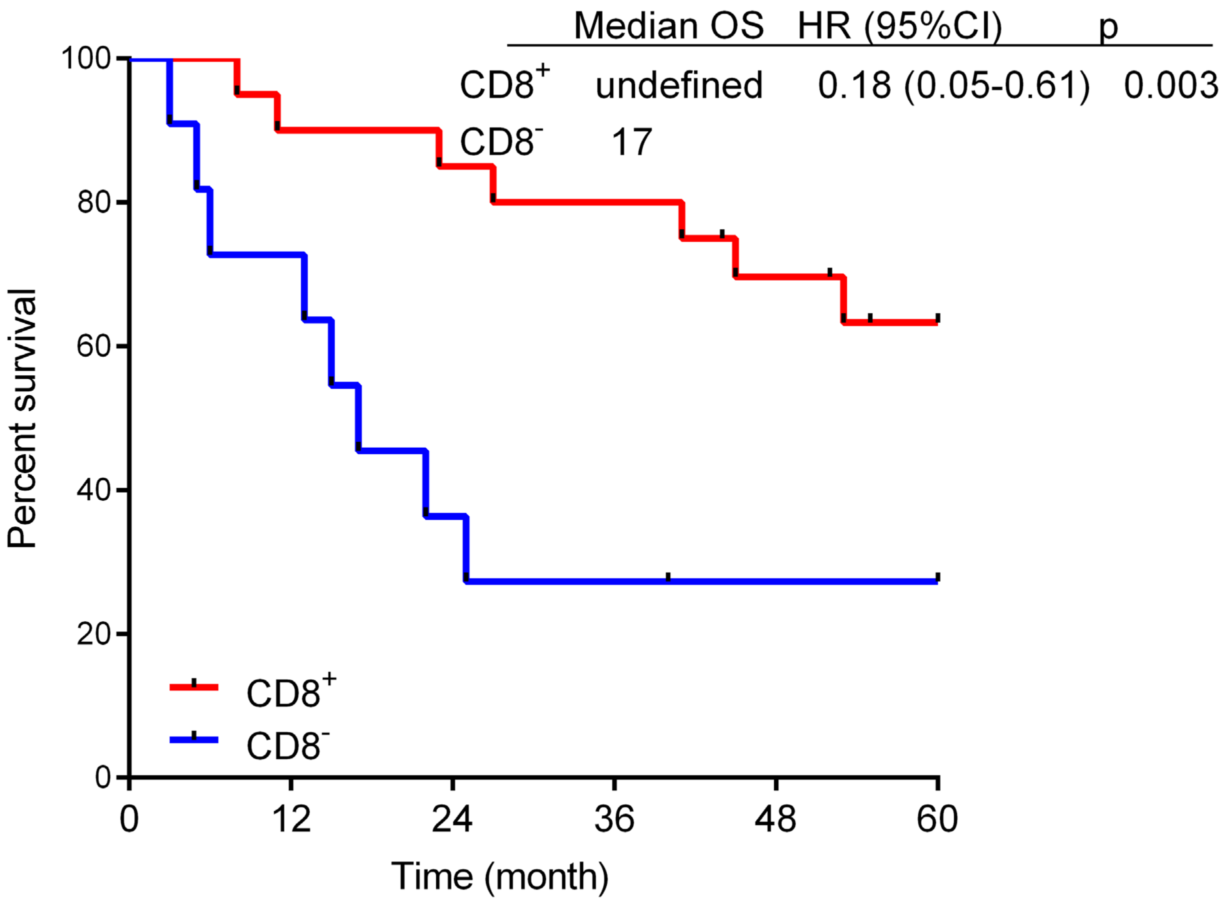

Supplementary Figure 2: Kaplan-Meier method log-rank testing for 5-year overall survival by CD8+ cell infiltration in Epstein-Barr virus-associated gastric cancer.

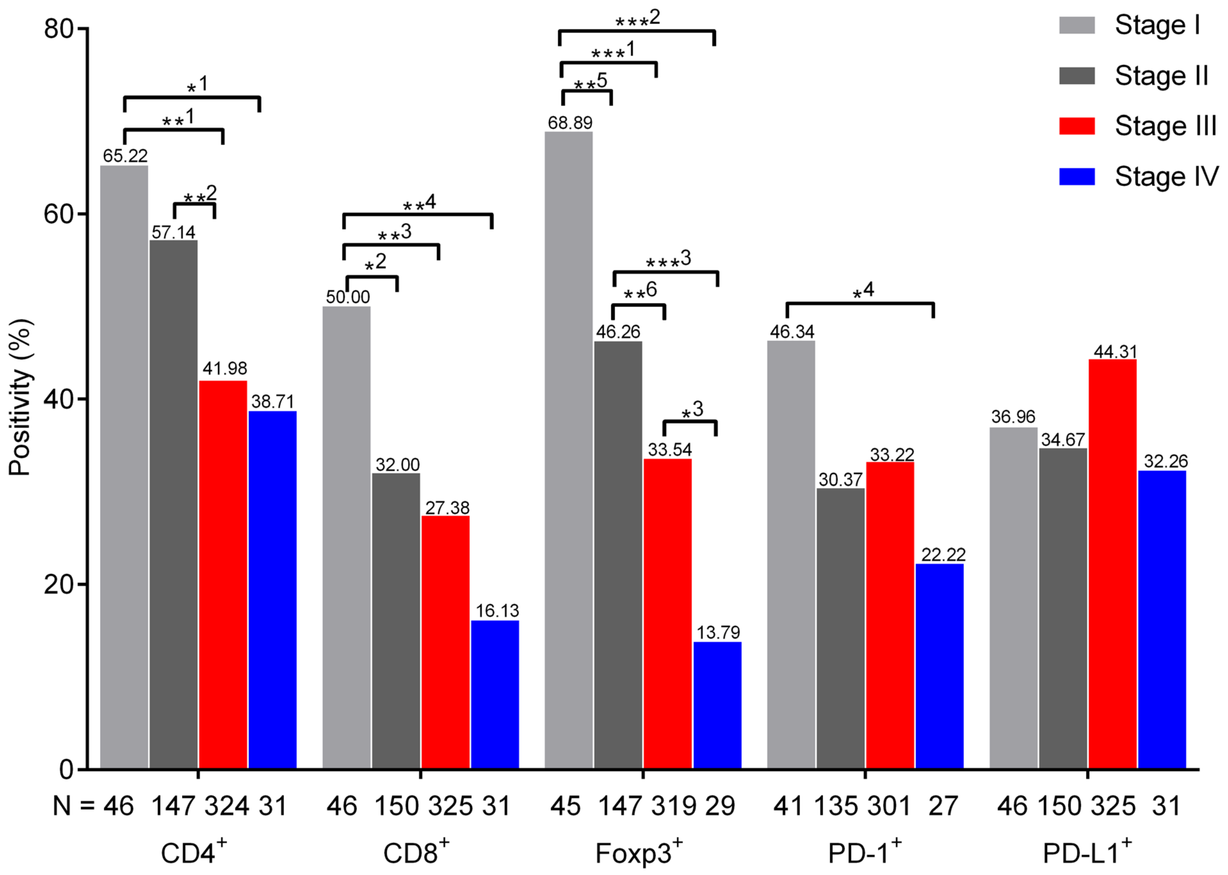

Supplementary Figure 3: The percentage of CD4⁺, CD8⁺, Foxp3⁺ cell infiltration and PD-1 and PD-L1 expression in different AJCC stages of gastric cancer. The number of patients in each subgroup was under X-axis and the positivity in different AJCC stages was labelled above each bar.

$*^1$p=0.022, $*^2$p=0.026, $*^3$p=0.029, $*^4$p=0.044;

$**^1$p=0.003, $**^2$p=0.002, $**^3$p=0.002, $**^4$p=0.002, $**^5$p=0.008, $**^6$p=0.008;

$***^1$p<0.001, $***^2$p<0.001, $***^3$p=0.001.

Bonferroni correction for multiple comparisons was applied, two side.

CD4, cluster of differentiation 4; CD8, cluster of differentiation 8; Foxp3, Forkhead box P3; PD-1, programmed death 1; PD-L1, programmed death ligand-1;

AJCC, American Joint Committee on Cancer

Supplementary Table 1: Clinicopathological characteristics of the patient populations

See Supplementary File 1

Supplementary Table 2: Clinicopathological characteristics of patients by different markers

See Supplementary File 2
