# Supplementary material for: Differentiated tumor immune microenvironment of Epstein–Barr virus-associated and negative gastric cancer: implication in prognosis and immunotherapy
Source: Oncotarget. 2017 May 16;8(40):67094–103. doi: 10.18632/oncotarget.17945 (PMC5620158; doi:10.18632/oncotarget.17945)
Supplement: Supplementary file 2 [file oncotarget-08-67094-s002.pdf]

Supplementary Table 1. Clinicopathological characteristics of the patient populations

	Complete cohort (n=571)		EBV status				
			Negative（n=540）		Positive (n=31)		
	N	%	N	%	N	%	p
Gender							0.111#
Male	407	71.3	381	70.6	26	83.9	
Female	164	28.7	159	29.4	5	16.1	
Age, year	571		540		31		0.049$
Median	59		59		54		
T stage							0.912#
1a	6	1.1	6	1.1	0	0	
1b	12	2.2	11	2.1	1	3.4	
2	67	11.6	65	12.4	2	6.9	
3	238	42.2	224	42.7	14	48.3	
4a	207	37.4	196	37.3	11	37.9	
4b	24	4.3	23	4.4	1	3.4	
N stage							0.091#
N0	112	20.4	107	20.6	5	17.2	
N1	102	18.6	92	17.7	10	34.5	
N2	137	25	132	25.4	5	17.2	
N3a	122	22.2	119	22.9	3	10.3	
N3b	76	13.8	70	13.5	6	20.7	
M stage							0.220#
M0	518	94.4	492	94.6	26	89.7	
M1	31	5.6	28	5.4	3	10.3	

AJCC							
Ia	14	2.6	13	2.5	1	3.4	0.370#
Ib	32	5.8	32	6.1	0	0	
IIa	54	9.8	49	9.4	5	17.2	
IIb	93	16.9	86	16.5	7	24.1	
IIIa	98	17.9	94	18.1	4	13.8	
IIIb	138	25.1	134	25.8	4	13.8	
IIIc	89	16.2	84	16.2	5	17.2	
IV	31	5.6	28	5.4	3	10.3	
Tumor location							0.181#
Upper 1/3	184	34.8	172	34.4	12	41.4	
Middle 1/3	84	15.9	78	15.6	6	20.7	
Low 1/3	211	39.9	205	41	6	20.7	
Upper 2/3 and whole	47	8.9	42	8.4	5	17.2	
Remnant	3	0.6	3	0.6	0	0	
Histology							0.493#
Adenocarcinoma	526	92.6	498	92.7	28	90.3	
Other types	42	7.4	39	7.3	3	9.7	
Differentiation							0.324#
High	109	56.7	106	19.5	3	9.5	
Moderate	138	24.2	131	24.3	7	22.8	
Low	324	56.7	303	56.2	21	66.7	

Abbreviations: EBV, Epstein-Barr virus; T, tumor; N, node; M, metastasis; N, number.

#Pearson $\chi 2$ or Fisher's exact test.

$Mann–Whitney U test.
