# Supplementary material for: Differentiated tumor immune microenvironment of Epstein–Barr virus-associated and negative gastric cancer: implication in prognosis and immunotherapy
Source: Oncotarget. 2017 May 16;8(40):67094–103. doi: 10.18632/oncotarget.17945 (PMC5620158; doi:10.18632/oncotarget.17945)
Supplement: Supplementary file 3 [file oncotarget-08-67094-s003.pdf]

Supplementary Table 2. Clinicopathological characteristics of patients by different markers

	CD4⁺ cell		CD8⁺ cell		Foxp3⁺ cell		PD-1		PD-L1	
	Absent N (%)	Infiltration N (%)	Absent N (%)	Infiltration N (%)	Absent N (%)	Infiltration N (%)	Positive N (%)	Negative N (%)	Positive N (%)	Negativ[illegible] N (%)
Gender	304	263	404	167	337	222	234	337	170	352
Male	225 (74.0)	180 (68.4)	289 (71.5)	118 (70.7)	237 (70.3)	162 (73.0)	170 (72.6)	237 (70.3)	119 (70.0)	251 (71.[illegible]
Female	79 (26.0)	83 (31.6)	115 (28.5)	49 (29.3)	100 (29.7)	60 (27.0)	64 (27.4)	100 (29.7)	51 (30.0)	101 (28.[illegible]
Age (year)	304	263	404	167	337	222	234	337	170	352
Median	29-86 (59)	21-87 (58)	28-82 (58)	21-87 (58)	28-87 (58)	21-86 (59)	21-87 (59)	28-87 (58)	26-87 (58)	21-86 ([illegible]
AJCC	286	262	387	165	330	210	223	329	165	338
Ia	4 (1.4)	10 (3.8)	8 (2.1)	6 (3.6)	4 (1.2)	9 (4.3)	4 (1.8)	10 (3.0)	4 (2.4)	8 (2.4[illegible]
Ib	12 (4.2)	20 (7.6)	15 (3.9)	17 (10.3)	10 (3.0)	22 (10.5)	13 (5.8)	19 (5.8)	15 (9.1)	14 (4.1[illegible]
IIa	19 (6.6)	34 (13.0)	38 (9.8)	17 (10.3)	24 (7.3)	30 (14.3)	18 (8.1)	37 (11.2)	13 (7.9)	33 (9.8[illegible]
IIb	44 (15.4)	50 (19.1)	64 (16.5)	31 (18.8)	55 (16.7)	38 (18.1)	34 (15.2)	61 (18.5)	27 (16.4)	61 (18.[illegible]
IIIa	52 (18.2)	45 (17.2)	64 (16.5)	34 (20.6)	61 (18.5)	35 (16.7)	45 (20.2)	53 (16.1)	21 (12.7)	71 (21.[illegible]
IIIb	80 (26.3)	58 (22.1)	110 (28.4)	28 (17.0)	91 (27.6)	46 (21.9)	55 (14.7)	83 (25.2)	47 (28.5)	79 (23.[illegible]
IIIc	56 (19.6)	33 (12.6)	62 (16.0)	27 (16.4)	60 (17.8)	26 (12.4)	44 (19.7)	45 (13.7)	32 (19.4)	51 (15.[illegible]
IV	19 (6.6)	12 (4.6)	26 (6.7)	5 (3.0)	25 (7.6)	4 (1.9)	10 (4.5)	21 (6.4)	6 (3.6)	21 (6.2[illegible]

Location	270	255	371	158	314	204	216	313	158	323
Upper 1/3	80 (29.6)	102 (40.0)	121 (32.6)	63 (39.9)	113 (36.0)	68 (33.3)	68 (31.5)	116 (37.1)	57 (36.1)	114 (35.[illegible]
Middle 1/3	37 (13.7)	46 (18.0)	62 (16.7)	22 (13.9)	44 (14.0)	39 (19.1)	47 (21.8)	37 (11.8)	27 (17.1)	41 (12.[illegible]
Lower 1/3	122 (45.2)	88 (34.5)	152 (41.0)	59 (37.3)	120 (38.2)	85 (41.7)	85 (39.4)	126 (40.3)	61 (38.6)	134 (41.[illegible]
Upper 1/3 and whole	29 (10.8)	18 (7.1)	33 (8.9)	14 (8.9)	34 (10.8)	12 (5.9)	14 (6.5)	33 (10.6)	13 (8.3)	31 (9.6
Remnant	2 (0.7)	1 (0.4)	3 (0.8)	0	3 (1.0)	0	2 (0.9)	1 (0.3)	0	3 (0.9)
Depth	301	261	401	165	335	219	233	333	168	349
Mucosa	1 (0.3)	2 (0.8)	1 (0.2)	2 (1.2)	1 (0.3)	2 (0.9)	0	3 (0.9)	1 (0.6)	2 (0.6)
Laminae muscularis mucosae	0	1 (0.4)	0	1 (0.6)	1 (0.3)	0	0	1 (0.3)	1 (0.6)	0
Submucosa	4 (1.3)	11 (4.2)	11 (2.7)	4 (2.4)	6 (1.8)	8 (3.7)	5 (2.1)	10 (3.0)	5 (3.0)	7 (2.0)
Muscular	23 (7.6)	39 (14.9)	40 (10.0)	23 (13.9)	23 (6.9)	38 (17.4)	28 (12.0)	35 (10.5)	23 (13.7)	36 (10.[illegible]
Serosa	151 (50.2)	142 (54.4)	215 (53.6)	79 (47.9)	171 (51.0)	122 (55.7)	129 (55.4)	165 (49.5)	78 (46.4)	183 (52.[illegible]
Subserosa	122 (40.5)	66 (25.3)	134 (33.4)	56 (33.9)	133 (39.7)	49 (22.4)	71 (30.5)	119 (35.7)	60 (35.7)	121 (34.[illegible]

Abbreviations: CD4, cluster of differentiation 4; CD8, cluster of differentiation 8; Foxp3, Forkhead box P3; PD-1, programmed death 1; PD-L1, programmed death ligand-1; N, number; AJCC, American Joint Committee on Cancer
